# Do Student Evaluations of University Reflect Inaccurate Beliefs or Actual Experience? A Relative Rank Model

**DOI:** 10.1002/bdm.1827

**Published:** 2014-06-13

**Authors:** Gordon D A Brown, Alex M Wood, Ruth S Ogden, John Maltby

**Affiliations:** 1 Department of Psychology, University of WarwickCoventry, UK; 2 Behavioural Science Centre, Stirling Management School, University of StirlingScotland, UK; 3 School of Natural Sciences and Psychology, Liverpool John Moores UniversityLiverpool, UK; 4 School of Psychology, Henry Wellcome Building, University of LeicesterLeicester, UK

**Keywords:** student satisfaction, rank-based judgement, context-dependence, NSS

## Abstract

It was shown that student satisfaction ratings are influenced by context in ways that have important theoretical and practical implications. Using questions from the UK's National Student Survey, the study examined whether and how students' expressed satisfaction with issues such as feedback promptness and instructor enthusiasm depends on the context of comparison (such as possibly inaccurate beliefs about the feedback promptness or enthusiasm experienced at other universities) that is evoked. Experiment 1 found strong effects of experimentally provided comparison context—for example, satisfaction with a given feedback time depended on the time's relative position within a context. Experiment 2 used a novel distribution-elicitation methodology to determine the prior beliefs of individual students about what happens in universities other than their own. It found that these beliefs vary widely and that students' satisfaction was predicted by how they believed their experience ranked within the distribution of others' experiences. A third study found that relative judgement principles also predicted students' intention to complain. An extended model was developed to show that purely rank-based principles of judgement can account for findings previously attributed to range effects. It was concluded that satisfaction ratings and quality of provision are different quantities, particularly when the implicit context of comparison includes beliefs about provision at other universities. Quality and satisfaction should be assessed separately, with objective measures (such as actual times to feedback), rather than subjective ratings (such as satisfaction with feedback promptness), being used to measure quality wherever practicable. © 2014 The Authors. *Journal of Behavioral Decision Making* published by John Wiley & Sons Ltd.

Understanding student satisfaction is of ever-increasing importance for reputational as well as educational reasons. In countries where surveys (e.g. the UK's National Student Survey (NSS) and Australia's Course Experience Questionnaire) ask the same questions of all students nationwide, the resulting ratings contribute to ranking tables that are highly publicized and widely consulted by prospective students (e.g. McDonough, Antonio, Walpole, & Perez, 1998), and institutions devote considerable resources to monitoring and improving satisfaction ratings.

Do such ratings accurately reflect objective student experience? Much of policy—at the level of both government and educational institutions—assumes that they do. Thus, it is typically assumed that publicizing the results of surveys of individual departments and universities can be used to drive improvements in the quality of the student experience. However, to the extent that subjective satisfaction ratings are driven by factors other than objective experience, strategies that will improve student satisfaction ratings may not be the same as strategies that will improve objective provision.

Here we apply cognitive models of context-based judgement to student satisfaction ratings. Our first aim is simply to show whether student satisfaction ratings will be subject to context effects in the same way as are other subjective judgements. While it is well understood that judgements of simple psychophysical and other unidimensional quantities are influenced by context (review can be found in the following discussion), students' judgements of their satisfaction with the educational provision they receive will reflect highly salient personal experience over a period often of years and hence may (as indeed is implicitly assumed by policy makers in a number of countries) be more absolute and less context dependent in nature. Our second and third aims are more concerned with theoretical issues. Most experimental studies of context effects focus on the immediate experimental context, examining for example how the judgement of a given stimulus is influenced by other stimuli alongside which it appears. Here we examine whether the context provided by the prior beliefs of an individual (e.g. possibly idiosyncratic beliefs about what happens in other universities) influences judgement in the same way as does experimentally provided context. Moreover, we use the data to examine whether purely rank-based models of context-based judgement, such as decision by sampling (DbS: Stewart, Chater, & Brown, 2006), can be extended to accommodate effects, such as those of the skew of the contextual distribution, that have previously been assumed to reflect other processes such as an influence of range (e.g. Parducci, 1965, 1995).

We begin by briefly reviewing previous research on the reliability and validity of student satisfaction ratings, emphasizing the distinction between different levels of analysis (e.g. whether the unit being evaluated is a course, instructor, department or whole institution). We then develop a specific model of context-based judgement. Three experimental studies that test the predictions of the model are then described, a revised and extended rank-based model is presented and implications for policy and practice are discussed.

## Reliability and validity of student satisfaction judgements

Student evaluations may take place either (i) *within* units such as departments that provide education to a group of students who largely share common experience (e.g. satisfaction with individual instructors or courses) or (ii) *between* units such as departments or whole institutions (e.g. satisfaction with a degree course) where there is often no direct personal experience of the alternatives (i.e. other departments and universities). Research on the reliability and validity of students' evaluations has reached different conclusions depending on the level of unit that is being evaluated (see, e.g. Marsh, Ginns, Morin, Nagengast, & Martin, 2011).

Research on students' ratings of particular courses and instructors, which has examined for example the relation between expected grades, subject interest and the resulting evaluations (e.g. D'Apollonia & Abrami, 1997; Marsh & Roche, 1997, 2000; Paulsen, 2002; Spooren & Mortelmans, 2006), has often shown encouraging results regarding reliability and validity (Marsh, 1984). Thus, student evaluations follow teaching improvements (e.g. Hativa, 1996; Marsh & Roche, 1993), notwithstanding other findings that evaluations are influenced by seemingly extraneous features of the educational situation (e.g. D'Apollonia & Abrami, 1997; Neath, 1996; Riniolo, Johnson, Sherman, & Misso, 2006) and that grading interpretations are context dependent (Wedell, Parducci, & Roman, 1989) (see also Sedlmeier, 2006). Students learn more from higher rated teachers (Cohen, 1981), although the relationship is complex and situation dependent with suggestions that evaluations are related to subjective rather than objective measures of learning (Clayson, 2009). Moreover, recent findings suggest that teaching in ways that increase evaluations of and performance in the taught class can reduce subsequent academic performance on other classes (Carrell & West, 2010).

In any case, reliability within a unit of analysis (e.g. evaluation of lectures within a single course, where students have a common context of experience) may not transfer to between-unit analysis (e.g. evaluation of a student's university compared with others, where the context of alternatives is not directly experienced by the students providing the ratings) (Cheng & Marsh, 2010). At the between-unit level, there is less evidence that satisfaction ratings straightforwardly reflect objective experience (see also Marsh et al., 2011). Cheng and Marsh found substantial disagreement amongst students in terms of their satisfaction with their overall educational experience, such that only around 2.5% of the variance in ratings could be attributed to real university-level differences. Such findings may reflect differing background contexts against which judgements are made. Previous research has not elicited quantitative measures of students' beliefs about the distribution of provision in institutions other than their own, but such measures—along with predictions of a well-specified model of exactly *how* contextual effects may operate—are necessary if the respective contributions of actual experience, on the one hand, and possibly erroneous beliefs about comparators, on the other hand, are to be distinguished. We develop such a model later.

## Cognitive models of educational satisfaction judgements

We build on a large body of cognitive research on how people make relative judgements—that is, on how a given stimulus is judged within a context (Vlaev, Chater, Stewart, & Brown, 2011). Although a given amount of provision (e.g. time to feedback) may be objectively measurable, forming a subjective judgement about the acceptability of the provision requires it to be compared with others. If student satisfaction ratings are necessarily context dependent, the questions naturally emerge of exactly how an amount of provision is compared with a context and whether errors and biases occur in these processes.

Several models of relative judgement processes exist. Here we focus on a widely applied descriptive account, range frequency theory (RFT: e.g. Parducci, 1965, 1995). According to RFT, judgements of items within a context depend on a weighted average of (i) how they *rank* in the context and (ii) where they fall within the overall *range* of the other stimuli. We refer to these as the ‘rank principle’ and ‘range principle’ respectively. Specifically, assume an ordered set of *n* contextual items [*x_1_*,*x_2_*,…..*x_i_*,….*x_n_*]. Then, if *M_i_* is the subjective psychological magnitude of *x_i_*,

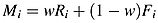
1where *R_i_* is the range value of stimulus *x_i_*

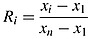
2and *F_i_* is the frequency value,^1^ or relative ranked ordinal position, of the item *i* in the ordered set

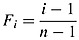
3

Range frequency theory and the rank and range principles have received substantial empirical support. Evidence came initially from psychophysics (Parducci, Calfee, Marshall, & Davidson, 1960; Parducci & Perrett, 1971) and subsequently in subjective judgements of (for example) sweetness (Riskey, Parducci, & Beauchamp, 1979), morality (Marsh & Parducci, 1978), temporal durations (Brown, McCormack, Smith, & Stewart, 2005), body image (Wedell, Santoyo, & Pettibone, 2005), attractiveness (Wedell, Parducci, & Geiselman, 1987), fairness (Mellers, 1982), personality, (Wood, Brown, & Maltby, 2011) and prices (Niedrich, Sharma, & Wedell, 2001; Niedrich, Weathers, Hill, & Bell, 2009). More recently, rank-based and RFT principles have been used to understand sensitivity to human fatalities (Olivola & Sagara, 2009), attitudes towards the riskiness of alcohol consumption (Wood, Brown, & Maltby, 2012), the effect of income on psychopathology (Wood, Boyce, Moore, & Brown, 2012) and individuals' satisfaction with both their wages (Brown, Gardner, Oswald, & Qian, 2008) and their life in general (Boyce, Brown, & Moore, 2010; Smith, Diener, & Wedell, 1989).

## Rank principle

What psychological process underpins the descriptive success of the rank principle? The DbS model (Stewart et al., 2006) suggests that judgements result from a series of binary ordinal comparisons of a to-be-judged target against other values in a mental sample. The DbS model emphasizes the role of comparison context retrieved from memory. In evaluating a feedback time of 15 days, for example, application of DbS suggests that a student would bring to mind a sample of other feedback times. These could be times mentioned by housemates studying other disciplines or by friends studying in other universities. If the mental sample contained 2 feedback times longer than 15 days, and 5 feedback times shorter, the subjective judgement of the 15-day feedback time would be determined by its relative rank position in the context, that is, 5/7. An implication is that different mental samples (reflecting differences in individuals' beliefs about provision elsewhere) would lead to different judgements about the same experienced provision; we tested this directly in Experiment 2.

## Range principle

The range principle suggests that educational provision will be judged relative to how it falls within the overall range of other amounts of provision in the relevant comparison context. For example, a given amount of provision, such as a 20-day wait until feedback is received, will be judged differently in a context that ranges from 10 to 30 days (where range position = .5) than in a context that ranges from 15 to 40 days (where range position = .2). A question of theoretical interest concerns the psychological process that gives rise to range effects. The DbS model (Stewart et al., 2006) offers a process-level interpretation of effects of relative rank. However, the model, as currently specified, predicts no effects of the range of a contextual distribution. After reporting the results of Experiment 1, we show that a purely rank-based model can be straightforwardly extended to account for effects of range.

## Practical implications

The interpretation of student satisfaction judgements, and the actions that will improve such judgements, depend on whether and how the context of comparison influences judgements. In particular, if students are evaluating their own universities in the context of possibly incorrect beliefs about what happens at other universities, satisfaction with provision may be influenced by factors other than objective quality of educational experience. Such a result would imply that student satisfaction and the objective quality of educational provision are different constructs and that the former cannot be straightforwardly used as a proxy for the latter. A possible implication is that—depending on policy objectives—the two constructs should be separately assessed.

## General methodology

Throughout, we examined three of the questions used in the UK's NSS. In 2010, over 250 000 students completed the 22-item NSS questionnaire (response rate 63%), and the majority of relevant UK institutions participated. In the NSS, and here, students stated their level of agreement with various statements using a five-point scale (*definitely agree*, *mostly agree*, *neither agree nor disagree*, *mostly disagree* and *definitely disagree*). In the studies here, these were coded 1 through 5 respectively such that higher numbers reflect higher dissatisfaction. The statements we examined were ‘Feedback on my work has been prompt’, ‘Staff have made the subject interesting’ and ‘I have been able to contact staff when I needed to’. These were selected to cover assessment, teaching and support domains respectively.

## EXPERIMENT 1: EFFECTS OF EXPERIMENTAL CONTEXT ON SATISFACTION JUDGEMENTS

**Figure 1:**
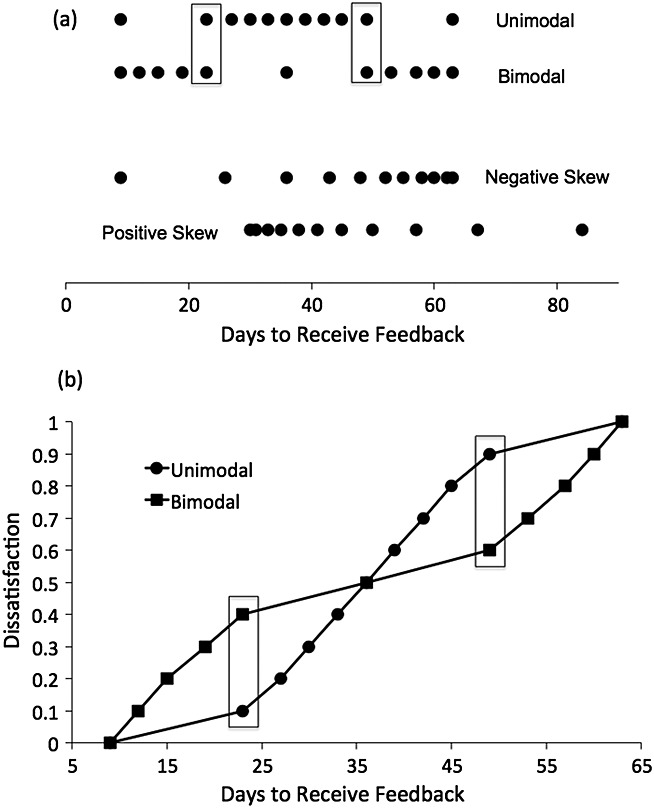
(a) Illustration of stimulus distributions constructed to test relative rank effects (upper two distributions) and range effects (lower two distributions). (b) Predictions of rank-based model for unimodal and bimodal distributions

Experiment 1 examined how the satisfaction associated with a given level of provision varied with the context of levels to be rated. The aim was to develop, using carefully controlled experimental distributions, an account of context effects on students' ratings that can then be extended to examine the effects of differing individual beliefs about real-world context in the second study. Following earlier work on RFT and DbS (e.g. Brown et al., 2008), we constructed pairs of distributions that would enable these hypotheses to be tested. The experimental logic is illustrated in Figure 1a, which shows the four distributions of feedback times (with the same numbers being used for other quantities as described later) used in this study. The numbers are also given in Table 1, with the common points highlighted in bold. The top two distributions (unimodal and bimodal) allow a clean test of the relative rank hypothesis. Consider the two values enclosed in rectangles. These are the same in each distribution (23 and 49 days), the same distance from the mean in each distribution and the same distance away from the shortest and longest feedback times. However, in the unimodal (top) distribution, 23 days represents the second shortest feedback time, whereas in the bimodal distribution, 23 days is the fifth shortest feedback time, and its relative ranked position is correspondingly higher. Thus, a feedback time of 23 days might attract higher satisfaction ratings in the unimodal distribution, where it is the second shortest. A similar argument applies for the feedback time of 49 days, which has a higher relative ranked position in the unimodal than in the bimodal distribution. Thus, any difference in the satisfaction ratings given to these two critical points unambiguously reflects an effect of rank. Figure 1b shows the ratings that would be given according to a purely rank-based model (Equation (3)), with the predicted crossover interaction for the critical points (which are again enclosed in rectangles).

**Table 1:** Levels of provision used in Experiment 1

Unimodal distribution	Bimodal distribution	Negatively skewed distribution	Positively skewed distribution
9	9	9	30
**23**	12	26	31
27	15	36	33
30	19	43	35
33	**23**	48	38
36	36	52	41
39	**49**	55	45
42	53	58	50
45	57	60	57
**49**	60	62	67
63	63	63	84

Numbers represent quantity to be rated (promptness of feedback, interestingness of instructors and ease of contact).

The second pair of distributions was included to test the range principle. The positive and negatively skewed distributions of feedback times have the same mean (46.5). However, if there is an effect of range, the average feedback time should seem longer in the negatively skewed condition, because more of the to-be-rated options are near the upper end of the scale. Although any individual item might seem lower in the positively skewed distribution, this is outweighed by the fact that more of the options are near the high end of the range in the negatively skewed conditions (Parducci, 1968). Because the relative rank position of each feedback time remains the same in both conditions, any effect of skew on average judgement will unambiguously reflect an effect of range. Participants also saw three other distributions of stimuli to examine hypotheses not examined in the present paper; the results from these are not reported here.

### Method

#### Participants

One hundred and fifty-two students (mean age 22 years; 59% female) took part in the experiment. Participation was voluntary and without payment.

#### Design and procedure

Distribution (unimodal, bimodal, positive and negative skew) was manipulated between subjects; quantity to be rated (promptness of feedback, interestingness of instructors and ease of contact) was manipulated within subjects. The values are shown in Table 1; these represented number of days to receive feedback, percentage of time lecturers made the subject interesting or percentage of time students were able to contact staff when they needed to. In each condition, participants rated, using the five-point scale described earlier, the extent to which they would agree with the relevant statement (e.g. ‘Feedback on my work has been prompt’) for each number (e.g. of days to receive feedback) if the amount of provision specified by the number applied to them. The wording of the instruction was for example (for the ‘provision of feedback’ condition) ‘if feedback on your work was returned to you in this number of days, to what extent would you agree that feedback on this piece of work was prompt?’ Items were presented as a column of numbers on the left-hand side of a single sheet of paper, with the five-point scale located to the right of each item. Half the participants received the numbers in ascending and half in descending order. Participants were tested individually.

### Results

We tested the key hypotheses in complementary ways. We first tested for effects of rank and range by fitting RFT to the data (choosing parameters to maximize the likelihood of the data given the model) and comparing the fit with that of a range-only model (to assess the rank effect) and a rank-only model (to assess the range effect). This approach allows exact likelihoods to be calculated for individual participants, allowing nested model tests of rank and range effects separately for each condition. Conventional statistical analyses were also undertaken where appropriate.

The model assumed that each discrete response from each participant reflected an underlying response tendency that was normally distributed on an underlying internal scale with standard deviation (SD) *s* and mean as predicted by RFT. *s* and *w* (cf. Equation (1)) were free parameters, as were scale endpoints. Here and in Experiment 2, we excluded participants who appeared to misunderstand the task or responded erratically (e.g. with an appropriately signed Kendall coefficient < |. 5| between responses and stimuli, or a response range ≤2). An average of seven participants' data were removed in the analyses.^2^

**Figure 2:**
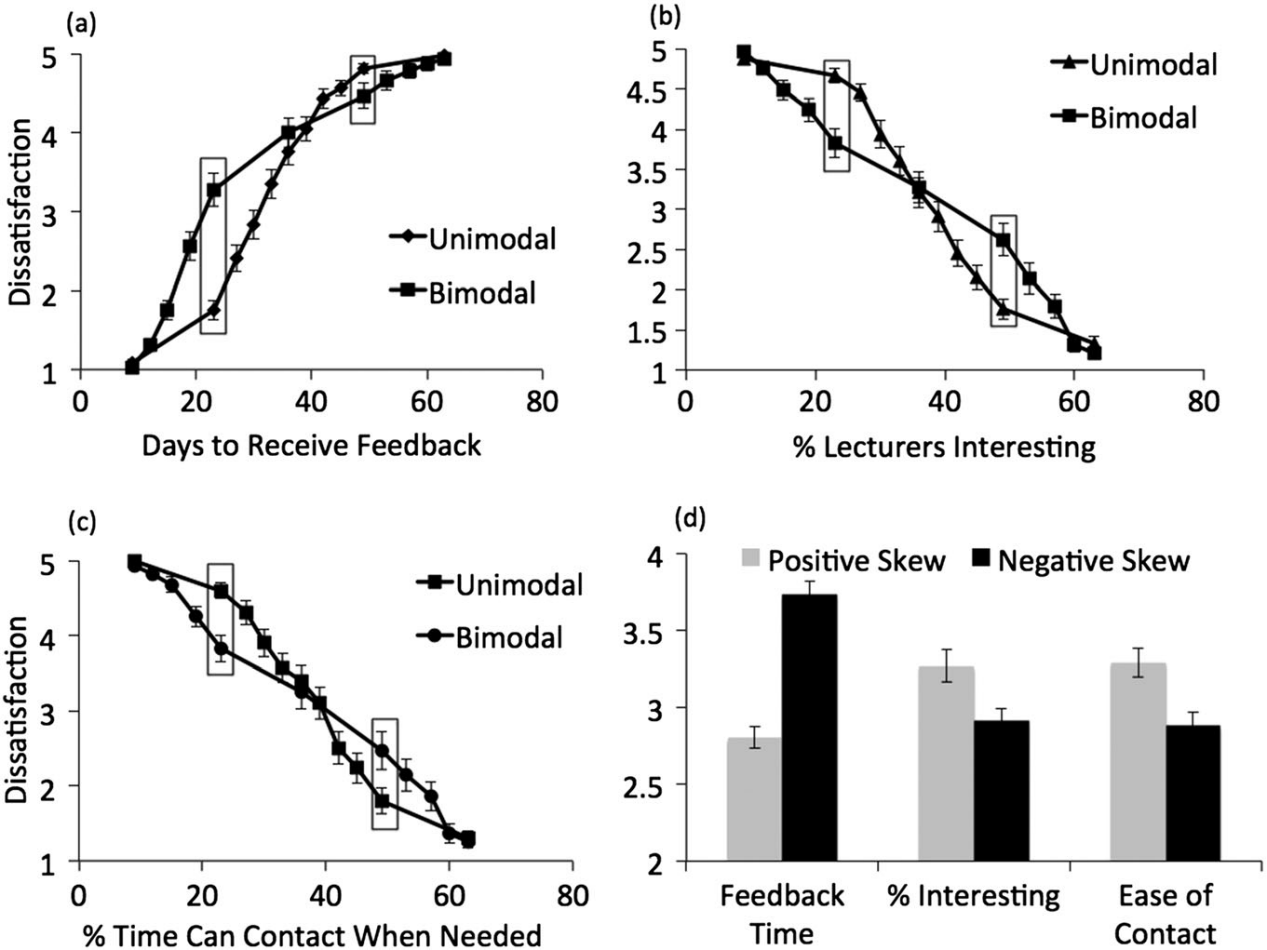
Results of Experiment 1. Panels (a) through (c) show dissatisfaction associated with unimodally and bimodally distributed feedback times (panel a), percentages of interesting instructors (panel b) and percentages of time staff could be contacted when needed (panel c). Panel d shows mean dissatisfaction to positively and negatively skewed distributions of the same quantities

Results are shown in Figure 2. The first three panels show the dissatisfaction associated with varying amounts of feedback time (panel a), instructor interestingness (panel b) and contact ease (panel c) for the unimodally and bimodally distributed quantities (test of rank effect). Panel d shows mean dissatisfaction in the positively and negatively skewed conditions. Common points are highlighted for panels a–c.

The first three panels show the characteristic crossover indicative of relative rank effects. Considering feedback time, for example, 23 days elicited less dissatisfaction in the unimodal (rank = 2) than in the bimodal condition (rank = 5). Conversely, 49 days elicited more dissatisfaction in the unimodal group (rank = 10) than in the bimodal group (rank = 7). Thus, the same feedback time caused different dissatisfaction depending on how it ranks within its context. Note that for the feedback condition, greater time results in more dissatisfaction, whereas in the remaining two conditions, higher contact ease or interestingness leads to less dissatisfaction.

Panel d shows results apparently consistent with a range effect: For the feedback condition, average dissatisfaction was higher in the negatively skewed distribution (where most of the feedback times were relatively long) with the expected reverse pattern for the other two conditions. Both model-based and conventional analyses confirmed these impressions as follows. The model-based analyses all involved nested model comparison. For all three outcome measures (feedback promptness, instructor interestingness and ease of contact), and for all four distributions (unimodal, bimodal, negatively skewed and positively skewed), we used maximum likelihood model fitting to compare RFT with rank-only and range-only models. Model comparison confirmed that RFT (containing both rank and range components) fit significantly better than either a range-only model (indicating a significant contribution of the ranked position of items) or a rank-only model (indicating a significant contribution of the range position of items). All model comparison statistics are shown in Table 2. Conventional analyses confirmed the findings as follows.

**Table 2:** Summary results of model comparison (Experiment 1)

Effects	Unimodal distribution	Bimodal distribution	Negatively skewed distribution	Positively skewed distribution
Promptness of feedback: Effect of rank	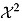 (37) = 33.2 *p* = .046	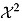 (32) = 89.5, *p* < .001	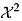 (32) = 218.5 *p* < .001	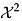 (31) = 89.3 *p* < .001
Promptness of feedback: Effect of range	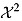 (37) = 176.5 *p* < .001	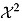 (32) = 55.6 *p* = .002	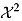 (32) = 60.3 *p* < .001	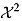 (31) = 286.2 *p* < .001
Interestingness of instructors: Effect of rank	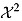 (33) = 20.0 *p* = .014	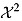 (29) = 70.2 *p* < .001	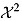 (28) = 73.2 *p* < .001	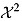 (26) = 103.4 *p* < .001
Interestingness of instructors: Effect of range	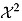 (33) = 207.3 *p* < .001	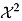 (29) = 56.7 *p* < .001	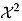 (28) = 180.3 *p* < .001	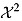 (26) = 211.6 *p* < .001
Ease of contact: Effect of rank	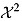 (30) = 31.0 *p* = .05	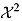 (28) = 102.2 *p* < .001	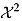 (32) = 61.6 *p* < .001	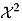 (26) = 106.2 *p* < .001
Ease of contact: Effect of range	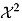 (30) = 163.5 *p* < .001	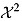 (28) = 41.8 *p* < .01	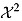 (32) = 267.3 *p* < .001	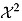 (26) = 151.1 *p* < .001

#### Promptness of feedback

An effect of rank was found, with a significant interaction between critical point (23-36-49) and distribution (unimodal versus bimodal): *F*(2,134) = 44.6, *p* < .001, partial *η*^2^ = .40. Range effects were tested by examining the difference between the mean judgements given in the positively and negatively skewed conditions. This difference was highly significant in the predicted direction, with higher overall dissatisfaction in the negatively skewed condition (*M* = 3.73, standard error (*SE*) = .09) than in the positively skewed condition (*M* = 2.80, *SE* = .07), *t*(61) = 8.24, *p* < .001, *d* = 2.11.

#### Interestingness of instructors

Analysis confirmed an effect of rank, with a significant interaction between critical point (values) and distribution (unimodal versus bimodal): *F*(2,120) = 30.2, *p* < .001, partial *η*^2^ = .34. A range effect was also found, with the difference between the mean judgements given in the positively and negatively skewed conditions being highly significant in the predicted direction—there was lower overall dissatisfaction in the negatively skewed condition (*M* = 2.91 *SE* = .08) than in the positively skewed condition (*M* = 3.27, *SE* = .11), *t*(52) = 2.68, *p* = .01, *d* = .74.

#### Ease of contact

There was a significant interaction between critical point (values) and distribution (unimodal versus bimodal): *F*(2,112) = 21.4, *p <* .001, partial *η*^2^ = .28, indicating an effect of rank. The difference between the mean judgements given in the positively and negatively skewed conditions was highly significant in the predicted direction, with lower overall dissatisfaction in the negatively skewed condition (*M* = 2.88 *SE* = .08) than in the positively skewed condition (*M* = 3.29, *SE* = .09), *t*(56) = 3.24, *p* = .002, *d* = .87.

### Discussion

The experiment found strong effects of context on student satisfaction judgements. The satisfaction associated with a particular feedback time, degree of interestingness or ease of contact was determined both by its relative ranked position within a context and by its location relative to the lowest and highest ‘anchor’ values provided by the context. The results confirmed that satisfaction ratings are highly context dependent as predicted by psychophysical models of judgement. Although we draw out wider implications in the General Discussion, we note here that the results of Experiment 1 may have practical implications for the relation between objective amounts of provision and the student satisfaction judgements associated with those amounts. The present results suggest that distortions could occur even when a given comparison context is largely shared by the students who are providing ratings (as when the units being evaluated are courses or instructors). More specifically, to the extent that students' satisfaction judgements are made in accordance with the principles of RFT, an improvement in objective provision time could in theory even reduce satisfaction if the improvement caused the distribution to become more negatively skewed. When the levels of analysis are larger (whole departments or institutions), the same effects may occur, but the additional consideration of non-shared comparison contexts also arises, and this possibility forms the topic of the next study.

### An extended model: skew effects from a rank-based process

What psychological processes might underpin the clear effects of the skew of contextual distributions that we have observed? As noted earlier, purely rank-based models of judgement predict no effect of skew. Here, however, we show that effects of skew may emerge if participants judge stimuli in terms of their relative ranked position within an inferred, rather than simply experimentally experienced, distribution. To illustrate, consider the task of estimating the ranked position of the height of a child within their classroom on the basis of observation of just the heights of the to-be-ranked child and two others. Suppose the height of the target child is 140 cm, whereas the other two observed heights are 138 and 155 cm. The relative ranked position of the target child within the sample is therefore .5. However, .5 would not be one's best estimate of the relative ranked position of the child if one also takes into account a strong prior assumption that heights will be normally distributed within a classroom. Given such a prior assumption, it is likely that there are several other unobserved children with heights in between 140 and 155 cm. A reasonable process would therefore be to estimate the parameters of a normal distribution from the observed sample of three children, and then calculate the relative rank of the 140-cm child within that inferred distribution. In this case, the parameters of the best-fitting normal distribution turn out to be *M* = 144 and *SD* = 9.3, and the relative ranked position of 140 within the associated cumulative distribution is .32—much less than the .5 estimate that would be given if background knowledge were not taken into consideration. Thus, background beliefs about prior distributions may lead to estimates of the relative ranked position of a stimulus that differ from its relative ranked position within an experimental sample. Note that it is not necessary to assume that participants actually compute the best-fitting distribution; a similar, although less precise, effect will occur if participants include previously experienced magnitudes (background knowledge) in their mental sample (Stewart et al., 2006).

**Figure 3:**
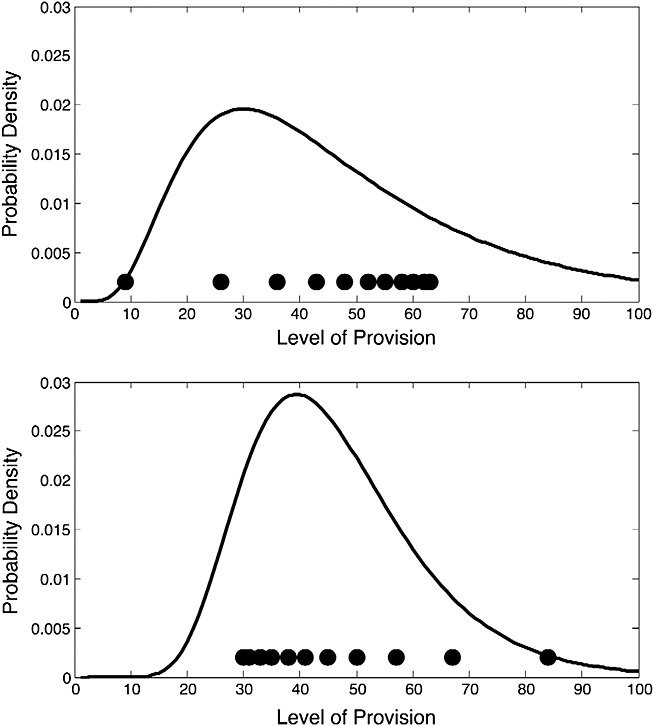
Best-fitting log-normal distributions to the negatively skewed (top panel) and positively skewed (bottom panel) stimuli used in Experiment 1

How much such an account shed light on the effects of skew that we have observed? Most distributions of natural quantities are positively skewed, and it is plausible that quantities such as times to receive feedback, or interestingness of lecturers, will follow a similar distribution. Here we illustrate using log-normal distributions. Figure 3 shows the best-fitting log-normal distribution to the positively and negatively skewed stimuli used in Experiment 1. It is evident that the curve that best fits the negatively skewed distribution of stimuli is flatter and more skewed to the right than is the curve that best fits the positively skewed distribution of stimuli. It is then possible to calculate the mean relative ranked position of each experimental stimulus within the inferred log-normal distributions. The relevant values turn out to be .55 for the negatively skewed stimuli and .48 for the positively skewed stimuli. In other words, if participants are sensitive to the relative ranked position of stimuli in an inferred distribution that reflects prior knowledge, effects of the skewness of experimentally presented stimuli can emerge. More specifically, the average relative rank (in the inferred distribution) of stimuli from the negatively skewed distribution is higher than is the average relative rank (in the inferred distribution) of stimuli from the positively skewed distribution. This is as observed experimentally.

Although such an account remains somewhat speculative, we suggest that it is at least possible that apparent effects of range (reflected in higher mean judgements for negatively rather than positively skewed distributions) might reflect purely rank-based processes operating on inferred distributions that may differ from experimentally experienced distributions as a result of augmentation from prior knowledge. In the next study, we address the issue of individual differences in prior beliefs directly.

It may be argued that Experiment 1—in which artificially constructed contextual distributions were provided experimentally and task demand characteristics may have been high—may not have captured the essence of real-world judgements in which the judgement context for a given student is unknown. Also, students were rating not their own experience but hypothetical quantities, where strong context effects might reflect task demands. When students must rate the quality of their department or university, as in the NSS, they may have little knowledge of what actually happens in other places. Furthermore, to the extent that students' beliefs about what happens in other places differ, they may not be equally satisfied with the same actual experience.

In order to test this possibility, it is necessary to know what each student believes about what happens outside their own institution, as these beliefs will provide the context of judgement. Experiment 2 examined this issue directly.

## EXPERIMENT 2: EFFECTS OF PRIOR BELIEFS ON SATISFACTION JUDGEMENTS

Experiment 2 (i) elicited students' beliefs about provision (e.g. promptness of feedback) available in other institutions and (ii) tested the prediction that their satisfaction can be predicted by the ranked position of their own amount of provision in the context of what they believe to happen elsewhere.

There are several ways of eliciting probability distributions (e.g. Lewandowsky, Griffiths, & Kalish, 2009; Manski, 2004); here students simply provided their estimates of different percentiles of the distribution. Participants were initially primed with the following statement (for the promptness of feedback item):

Different universities take a varying number of days to provide feedback for assessed work. We are interested in how quickly you think that universities typically provide feedback for assessed work in the UK. Of course, we do not expect you to know this information exactly; we are interested in your best estimate even if it is a guess. Please answer the questions below.

They were then asked questions of the following form:

Imagine there are 100 universities in the UK. In days, how quickly would a university have to typically provide feedback on assessed work to be faster than 90 out of these 100 universities (i.e. to rank in the top 10%)? ___

where the percentiles asked about were 90, 80, 70, 60, 50, 40, 30, 20 and 10.

**Figure 4:**
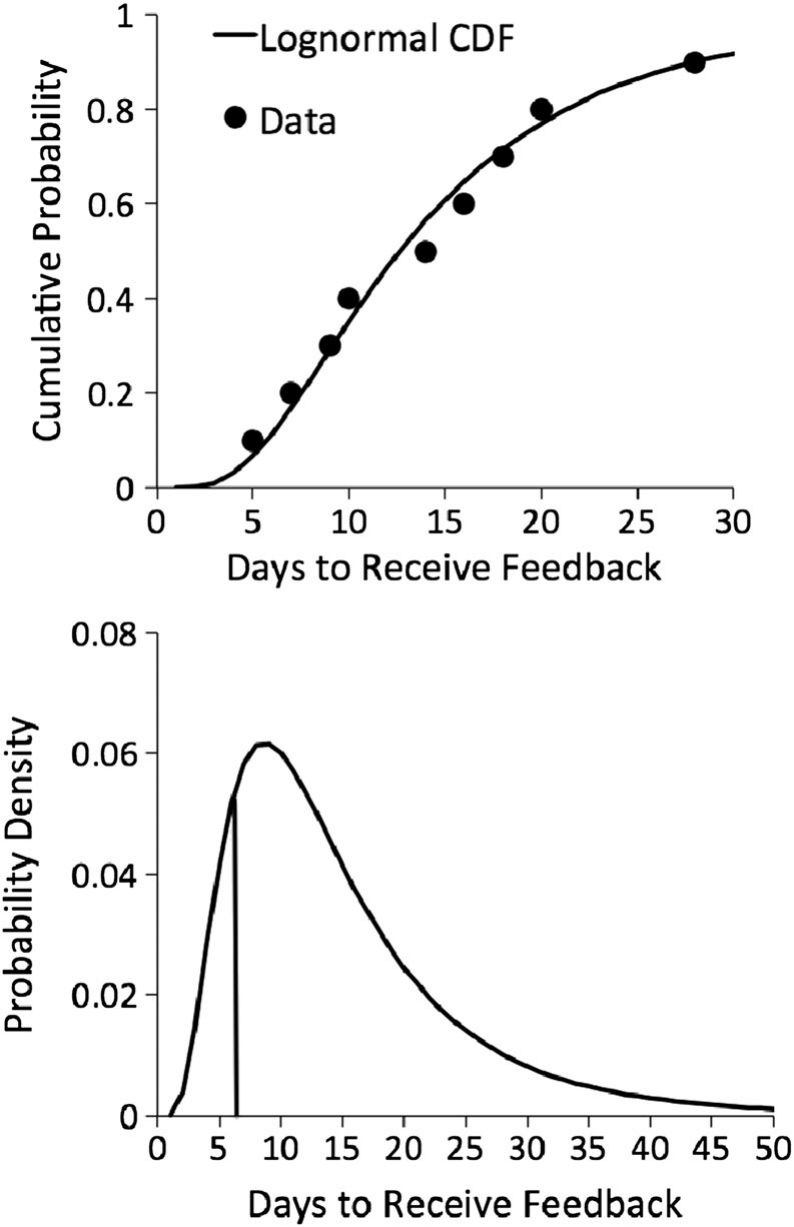
Illustration of distribution-elicitation methodology (see text for details)

We then fitted a cumulative distribution to each participant's responses. Figure 4a shows a cumulative log-normal fit to the data from one participant in the study described later. This participant believed that 50% of other students received feedback in 13 days or fewer. Having thus estimated each participant's beliefs about provision at other institutions (Figure 4b; henceforth dubbed ‘subjective distribution’), we calculated the mean of the distribution (‘subjective mean’) and the relative ranked position of the student's own experience (‘subjective rank’) within the distribution. This student's own feedback was received in 7 days on average. Thus, the student in question believed that about 17% of other students received feedback more quickly than they did themselves.

Our aim was to examine the extent to which students' satisfaction will be predicted not (or not only) by their actual amount of provision but rather by the ranked position of their own provision within their subjective distribution. It is also of theoretical importance to exclude the possibility that students judge their own amount of provision relative to what they believe the *mean* amount of provision to be, and to this end, we also examined whether the ranked position of amount of provision (within the subjective distribution) independently predicted satisfaction when the mean of the subjective distribution was also used as a predictor.

### Method

#### Participants

One hundred and seventy-one students (69% female; average age 20 years) participated in fulfilment of course requirements. All were undergraduates; most were studying psychology. None had participated in Study 1.

#### Design and procedure

Students completed Internet questionnaires at a time of their choosing. The task was first explained (e.g. ‘Different universities take varying amount of days to provide feedback for assessed work. There are 132 universities in the UK. We are interested in how quickly you think that these universities provide feedback for assessed work.’) Participants then provided the percentile estimates. Participants then stated the average feedback time they had experienced, the percentage of their lecturers who are interesting and the percentage of time they could contact staff when they needed to. Students also stated their satisfaction with feedback promptness, staff interestingness and ease of contact (using NSS wording).

### Results

There were three steps to the analysis of each of the three questions. First, we estimated each participant's cumulative distribution function (using either a log-normal or a linear function according to which fitted best; most participants produced data consistent with a positively skewed distribution of the relevant quantities). Second, we calculated the mean of each participant's subjective distribution function (‘subjective mean’) and the relative rank position of the participant's own experience (‘subjective rank’) within it. Finally, we used regression (Polytomous Universal Model (PLUM); logistic link function) to predict satisfaction ratings from subjective mean and rank along with other predictors as specified later.

Participants' beliefs about the range of provision offered in other universities were highly variable and inaccurate. For example, 10% of students thought that the median feedback time elsewhere was 40 days or longer; 10% thought it was 9 days or fewer. However, only 2 of 168 participants said that their own normal feedback time was longer than 40 days. Ten per cent of students thought that the median percentage of interesting teaching staff was 25% or less, whereas 10% believed it was more than 60%, and 10% of students thought that the median percentage of time that could be contacted when needed was 25% or less, whereas 10% believed the figure was more than 70%.

#### Satisfaction with promptness of feedback

Data from two participants were removed on the criterion for the Kendall coefficient relating responses to stimuli (all other values were >.85). There was considerable variation both in statements of own feedback time (range: 2 to 56; *SD* = 7.8) and in satisfaction with it (range: 1 to 5; *SD* = .94). Initial Pearson correlations revealed that dissatisfaction with feedback time was significantly correlated with own feedback time (*r* = .17; *p* = .028), with subjective rank (*r* = .40, *p* < .001) and with subjective mean (*r* = −.18, *p* = .02). The satisfaction variable may be more conservatively interpreted as an ordinal measure; non-parametric correlations (Kendall's *τ*) revealed essentially the same pattern except that the association between dissatisfaction with feedback time and own feedback time was reduced to non-significance (*τ* = .092, *p* = .138).

Regressions were then conducted to predict students' agreement that their ‘feedback is prompt’ from (i) gender, (ii) year of study, (iii) stated own feedback time, (iv) subjective rank and (v) subjective mean. Parameter estimates for the variables of theoretical interest (iii–v) are shown in Table 3. There were no statistically significant independent effects of gender or year of study, and the coefficients for these variables are not reported. An initial regression with predictors (iii) and (iv) found that stated own feedback time did not independently predict dissatisfaction, whereas subjective rank did (Table 3). A second regression was conducted with subjective mean as an additional predictor; subjective mean did not account for additional variance and did not remove the effect of subjective rank (Table 3). Thus, student satisfaction with feedback time was predicted solely by subjective rank, with, notably, no additional contribution from students' own feedback time.

**Table 3:** Regression coefficients from analysis of Experiment 2

	Feedback time				
Regression		Coefficient	Standard error	Wald	*p*
1	Stated time	.021	.020	1.003	.317
	Subjective rank	3.165	.682	21.558	.000
2	Stated time	.019	.031	.362	.548
	Subjective rank	3.238	1.192	7.377	.007
	Subjective mean	.001	.024	.006	.940
Nagelkerke pseudo-*R*^2^ = .18					
	Interestingness				
1	Stated %	−.010	.011	.857	.355
	Subjective rank	−3.294	.891	13.673	.000
2	Stated %	−.002	.020	.008	.929
	Subjective rank	−3.913	1.538	6.467	.011
	Subjective mean	−.010	.020	.238	.626
Nagelkerke pseudo-*R*^2^ = .30					
	Ease of contact				
1	Stated %	−.027	.011	6.379	.012
	Subjective rank	−2.003	1.090	3.379	.066
2	Stated %	−.032	.021	1.963	.136
	Subjective rank	−1.641	1.892	.753	.386
	Subjective mean	.005	.023	.051	.822
Nagelkerke pseudo-*R*^2^ = .23					

#### Satisfaction with instructor interestingness

Data from all but one participant were retained, all showing a Kendall coefficient of .85 or greater, and correlations and regressions were undertaken as reported earlier. There was wide variation both in statements about percentage of their instructors who were interesting (range: 1 to 90; *SD* = 23.3) and in dissatisfaction with that percentage (range: 1 to 4; *SD* = .84). Initial Pearson correlations revealed that dissatisfaction was significantly correlated with percentage of interesting lecturers (*r* = −.40; *p* < .001), and with subjective rank (*r* = −.48, *p* < .001), but not with subjective mean (*r* = .06, *p* = .47). Non-parametric correlations (Kendall's *τ*) revealed the same pattern.

Regressions were carried out as for dissatisfaction with promptness of feedback; parameter estimates are shown in Table 3. There were no effects of gender or year of study. The first regression (with percentage of interesting instructors and subjective rank as the predictors) found that including subjective rank completely removed the effect of percentage of interesting instructors on dissatisfaction. Subjective rank was itself a strong predictor. The pattern remained unchanged when subjective mean was included as a third predictor. In summary, agreement with the statement that lecturers are interesting was predicted solely by subjective rank, with no additional contribution from participants' own experience.

#### Satisfaction with ease of contact

Data from 14 participants were removed—in the majority of cases because some percentile questions were left unanswered. Correlations and regressions were then undertaken as done earlier. There was wide variation both in statements about the percentage of time students could contact instructors when they needed to (range: 5 to 100; *SD* = 24.3) and in satisfaction with that amount of provision (range: 1 to 4; *SD* = .80). Initial Pearson correlations revealed that dissatisfaction was significantly correlated with percentage of time contact was possible (*r* = −.45; *p* < .001), and with subjective rank (*r* = −.42, *p* < .001), but not with subjective mean (*r* = .05, *p* = .52). Non-parametric correlations (Kendall's *τ*) revealed the same pattern.

Regressions were then undertaken as done earlier; parameters are shown in Table 3. Again, there were no effects of gender or year of study. In contrast to the previous two analyses, the first regression found an effect of absolute percentage of contactability and a marginally significant effect of subjective rank, but in the second regression, in which subjective mean was also included, no variables independently predicted dissatisfaction with ease of contact.

### Discussion

The predictions of the rank-based accounts were largely confirmed. For feedback promptness and lecturers' interestingness, students' satisfaction with their own amount of provision was predicted only by the ranked position that their own experience occupies within what that student believed to be the distribution elsewhere. Indeed, the students' stated own amount of provision accounted for no significant additional variance and nor did subjective means. The fact that subjective means accounted for no independent variance provides some evidence against a simple adaptation-level account according to which students' satisfaction with provision would be determined by how the level of provision relates to what the mean level of provision is believed to be.

The result has both theoretical and practical implications. At a theoretical level, the study suggests that the same rank-based principles operate on retrieved contextual distributions as have been found to describe judgements in purely experimental contexts. The results also speak to the issue of whether global context (e.g. information about the category to which to-be-judged items belong) may influence judgement (Pettibone & Wedell, 2007). Pettibone and Wedell describe a model in which presentation of an item may lead to retrieval of both recent and category-relevant information, and they find stronger contextual effects of recent rather than category-relevant items, although with categorical context being more important when comparison items must be retrieved from memory. The present data add to the evidence that context retrieved from memory can influence judgements when, as here, the judgement task requires it.

In terms of implications for educational practice, the results suggest that variation in incorrect beliefs about what happens elsewhere, in combination with own experience of provision, will determine satisfaction judgements. We note that this point applies primarily when provision is being evaluated at the levels of whole departments or institutions, as it is in such cases that there is less common experience and hence more room for inaccurate beliefs to persist. Thus, expressed satisfaction could change either as a result of objective improvements in provision (which will lead to improved ratings provided that beliefs about the comparison context, however inaccurate, remain unchanged) or as a result of changing beliefs about the amount of provision elsewhere.

## EXPERIMENT 3: EFFECTS OF EXPERIMENTAL CONTEXT ON INTENTION TO COMPLAIN

Whereas the first two experiments examined how experimentally provided contexts (Experiment 1) and retrieved beliefs about context (Experiment 2) influenced satisfaction judgements, Experiment 3 examined whether the context-based model could be extended to intention to complain about provision. We hypothesized that context would influence—in predictable ways—the objective amount of provision that would lead subjects to complain. Specifically, it was predicted that stated intention to complain about (for example) promptness of feedback would be associated with a longer hypothetical feedback time in the context of a negatively skewed distribution of feedback times (in which most feedback times are towards the upper end of the range of experienced feedback times).

### Method

#### Participants

Eight-five students (67% female; average age 22 years) participated in fulfilment of course requirements. All were undergraduates; most were studying psychology.

#### Design and procedure

Participants viewed positively or negatively skewed distributions of provision amounts for each of the three domains examined in Experiments 1 and 2 (promptness of feedback, ease of contactability and interestingness of lecturers). They were provided instructions of the following form, with appropriate changes for the different domains:

After submitting work for assessment, it can take a varying amount of time to receive feedback. Please imagine that you handed in 11 pieces of important assessed coursework over the course of your degree. In the table below, you will see the time that it took for these 11 pieces of coursework to be returned to you with comments on how you may improve the work in future. For each of these pieces of coursework, we are interested in whether you would complain if it took this length of time for the work to be returned. We are interested in complaints such as following: formal procedures, writing to your personal tutor, speaking to your student representative, or e-mailing or speaking to another relevant member of staff. Please indicate next to each of the feedback times whether you would complain, by selecting the relevant option.

The 11 numbers representing different amounts of provision were 9, 10, 12, 14, 17, 20, 24, 29, 36, 46 and 63 (positive skew condition) or 9, 26, 36, 43, 48, 52, 55, 58, 60, 62 and 63 (negative skew condition); data from an additional distribution were collected but were not analysed. The numbers represented either number of days taken to receive feedback, percentage of time instructors' lectures were interesting or percentage of time instructors could be contacted when needed.

Items (different amounts of provision) were presented in a column on the left-hand side of a single sheet of paper, with response options (‘would you complain? Yes/No’) located to the right of each item. Half the participants received the numbers in ascending and half in descending order. Participants were tested individually.

For each amount of provision, students stated whether or not they would complain if they received that amount. According to the contextual account, the distribution of possibilities should strongly influence the absolute amount of provision associated with the stated intention to complain. For example, it was predicted that a greater feedback time would be needed to elicit complaint in a negatively skewed distribution of feedback times (because in such a distribution, most feedback times are already relatively lengthy).

### Results

Data from a minority of participants who stated they would not complain for any level of provision listed, or would complain for every level of provision or whose responses suggested they would complain for better amounts of provision (one participant) were excluded. This led to the retention of 74, 65 and 79 participants in the feedback time, interestingness and contactability conditions respectively. As predicted, students stated that they would complain at a much lower level of provision when that provision was positively skewed (feedback time) or negatively skewed (instructor interestingness and contact ease).

Unwillingness to complain ranged from 2% (contact ease) to 23% (instructor interestingness). The feedback time that would attract complaint was greater in the negatively skewed condition (*M* = 47.4, *Standard Error of the Mean* = 1.38) than in the positively skewed condition (*M* = 31.5, *Standard Error of the Mean* = 2.25; *t*(72) = 6.18, *p* < .001, *d* = 1.46). The percentage of instructor interestingness that would not lead to complaint was also greater in the negatively skewed condition (*M* = 42.3, *Standard Error of the Mean* = 2.09) than in the positively skewed condition (*M* = 24.1, *Standard Error of the Mean* = 1.88; *t*(63) = 6.50, *p* < .001, *d* = 1.64). Similarly, absence of complaint was associated with a higher percentage of times that instructors could easily be contacted in the negatively skewed condition (*M* = 42.0, *SE* = 1.93) than in the positively skewed condition (*M* = 25.44, *Standard Error of the Mean* = 1.49; *t*(77) = 6.85, *p* < .001, *d* = 1.56).

The preceding analyses show that context influences the level of provision that leads to a stated intention to complain. Further analysis, not reported in detail here, found that the within-context ranked position of the level leading to complaint was significantly higher in the positively skewed condition, consistent with there being an absolute as well as a relative component to judgement.

### Discussion

The predictions of the contextual account were again confirmed. Students' stated willingness to complain about the amount of their provision was strongly influenced by the distribution of possible amounts of provision under consideration, as predicted by cognitive models of judgement. As with the conclusions from Experiment 1, this effect could operate when satisfaction is expressed regarding any level of provision (i.e. from individual instructors to whole institutions) as the effect does not rely on incorrect beliefs about provision elsewhere. In practical terms, the result again suggests that care should be taken to ensure an appropriate distribution of, as well as mean amount of, provision.

## GENERAL DISCUSSION

We applied a model of context-based judgement to students' educational satisfaction ratings. We found considerable evidence that students' rated satisfaction with their overall university experience is strongly context dependent. The satisfaction associated with a given amount of provision (e.g. feedback on coursework being provided after a particular number of days) depends not just on the objective amount of provision but on how the provision relates to a context of other remembered, experienced or incorrectly believed amounts of provision.

In Experiment 1, we found that satisfaction ratings were influenced predictably by the context of to-be-evaluated options. The effects observed in Experiment 1 were large in magnitude, with for example the mean dissatisfaction with feedback promptness increasing from 2.8 to 3.7 (on a five-point scale) when the distribution of feedback times was negative rather than positive, even though the mean feedback time was the same in both distributions. In Experiment 2, assumed to approximate the judgement processes occurring in real life more closely, we found (i) that students' beliefs about levels of provision in other universities were highly variable and (b) that students' rated satisfaction with their own amount of provision was strongly influenced by their beliefs about what happened elsewhere. Experiment 3 showed that stated willingness to complain was also influenced by context as predicted by cognitive models of judgement. Finally, we have argued that rank-based models of judgement can account for effects of the skew of contextual distributions (i.e. effects that have previously been assumed to implicate range effects).

What are the implications for policy and practice? The strong effects of context of satisfaction judgement suggest that objective quality and subjective satisfaction are different things and should be assessed accordingly. Quality and satisfaction are both legitimate policy targets, but as far as possible, one should not used be used as a proxy for the other. Contextual influences may emerge whatever the level being judged (instructors, courses and institutions), as context-based judgement will occur whether or not students are correct in their beliefs about the appropriate comparison context. However, the practical consequences—for reliability, validity, policy and practice—differ across levels of analysis. First, we consider judgements about individual courses or instructors when raters largely share a common context (e.g. when the students who are providing the ratings attend the same courses and are exposed to the same set of instructors). In this case, we may expect the ratings to be generally reliable, because context effects will be largely the same for all raters. Furthermore, judgements will generally be valid to the extent that the range and distribution of provision amounts do not change substantially. However, validity will be compromised when the skew of the distribution changes (e.g. if one very good lecturer is introduced) or if every amount of provision changes by a constant. In both cases, changes in objective amounts of provision might not be accompanied by the expected changes in subjective satisfaction ratings.

Second, we consider judgements about general levels of provision in departments or institutions when raters largely share a comparison context. In this case, as with the first, contextual effects need not prevent reliability when context is shared. However, distributions of provision that have the same objective mean but different skews may be associated with different judgements (as in Experiment 1). Institutions concerned to improve their evaluations may be well advised to consider the distribution of provision amounts experienced by students, not just their mean amount. Furthermore, organizations wishing to improve the objective quality of student experience should focus on objective measurements wherever possible, rather than relying on student satisfaction ratings. Sometimes, the use of objective measures will not be feasible (e.g. in the evaluation of ‘interestingness of lecturers’). In other cases, however, such as the time taken to receive feedback, objective measures could be used instead if the cost of collecting them is not prohibitive. Indeed, the replacement of subjective with objective measures could be beneficial for other reasons: As noted by Armstrong (2012), the very fact that consumers know that they will be required to produce satisfaction judgements may in some contexts reduce their reported satisfaction (Ofir & Simonson, 2001).

Third, we consider judgements of whole departments or institutions when raters may have different beliefs about the comparison context. In this case, and as confirmed by the results of Experiment 2, raters' differing (and often inaccurate) beliefs about provision elsewhere will strongly influence their judgements, which cannot therefore be interpreted as indicators of objective amounts of provision. However, (i) averaging over raters may mitigate such effects, and (ii) improvements in objective experience should lead to improvements in subjective satisfaction ratings if beliefs about provision elsewhere do not change.

In summary, students' satisfaction with general aspects of the course experience is heavily influenced by context and is, for some types of provision, driven largely by their (often erroneous) beliefs about what happens in other institutions. In particular, subjective ratings are influenced by context in just the way predicted by simple psychophysical models of judgement. In processing terms, the results are consistent with participants using a decision by sampling strategy (Stewart et al., 2006).

Limitations of the study include the collection of data from only a single institution in Experiment 2, although a wide range of stated experienced provision was found in all cases and that this limitation seems unlikely to explain why strong relative rank effects were found (although it might contribute to the absence of other effects). It is also difficult to exclude the possibility that the elicitation of distributions may have caused participants to focus more on external context than they otherwise would have, although such an effect would not explain the observation that relative rank, rather than subjective mean, predicted satisfaction ratings. Moreover, the operationalization of the ‘interestingness’ question as ‘percentage of interesting teaching staff’ in Experiment 2 treats interestingness as a binary rather than continuous variable and hence may relate only indirectly to the NSS question wording.

Finally, it would clearly be wrong to conclude that genuine improvements in provision would have no effect on reported satisfaction in a real-world setting. Crucially, however, changes in both (i) the provision provided to students and (ii) students' beliefs about the comparison context need to be considered. For example, satisfaction would not improve if feedback times are reduced, but at the same time, students come to believe that other institutions provide feedback in a shorter time than the students had previously assumed. A given lecturer could receive lower satisfaction despite having improved his or her course if colleagues improved their courses to a greater degree. Similarly, if other universities improve provision faster than a student's own (or are incorrectly believed to have done so), satisfaction may decrease despite provision having improved. The resulting ‘arms races’ may be an intended or unintended consequence of a policy focus on student satisfaction; provision may improve rapidly through competition even though satisfaction does not increase in parallel. Such competition may be valuable for the objective student experience, but carries a danger of instructor demoralization.

In conclusion, the fact that student satisfaction ratings are highly relative need not mean that they will always be unreliable or invalid. However, our results have practical implications concerning targets for interventions. In particular, it matters whether objective experience or subjective satisfaction is the target of change. If the intention is to improve objective amounts of provision, then objective measures (such as actual times to feedback), rather than subjective ratings (such as satisfaction with feedback promptness), should be used to measure quality wherever practicable. If the primary aim is, in contrast, to improve student satisfaction rather than objective amounts of provision, then contextual determinants of satisfaction such as students' perceptions of ‘what is normal’ need to be taken into account.

## Footnotes

1 The use of the term ‘frequency’ in RFT reflects the fact that original tests of the model created skewed distributions by varying the frequency with which stimuli were presented rather than, as in the present paper, the spacing of once-presented items.

2 There was no evidence that this procedure could change the qualitative pattern of results.
